# Associations of family socioeconomic indicators and physical activity of primary school-aged children: a systematic review

**DOI:** 10.1186/s12889-024-19174-6

**Published:** 2024-08-19

**Authors:** Alexandra Ziegeldorf, Daniel Schoene, Alisa Fatum, Katharina Brauer, Hagen Wulff

**Affiliations:** 1 Institut for Execise and Public Health, Faculty for Sports Science, University Leipzig, Jahnallee 59, 04109 Leipzig, Germany; 2 Department of Clinical Gerontology and Geriatric Rehabilitation, Robert Bosch Hospital, Auerbachstr. 110, 70376 Stuttgart, Germany; 3 Institute of Radiology, University Hospital of Erlangen, Friedrich Alexander University, Erlangen-Nürnberg, Maximiliansplatz 1, 91054 Erlangen, Germany; 4 Institut for Sports Pedagogy, Faculty for Sports Science, University Leipzig, Jahnallee 59, 04109 Leipzig, Germany

**Keywords:** Children, Socioeconomic status, Education, Occupation, Income, Physical activity

## Abstract

**Background:**

Family socioeconomic indicators (education, occupation, and household income) are key determinants influencing children’s physical activity (PA). This study aims to systematically review the current research about the association between family socioeconomic indicators and PA among primary school-aged children and to quantify the distribution of reported associations by childs’ and parents’ sex and according to analysis and assessment methods.

**Methods:**

A systematic literature research in multiple scientific databases (MEDLINE via PubMed, Web of Science, ScienceDirect, SPORTDiscus and ERIC) was performed for literature published between 1st January 2010 and 31st March 2022. Only studies reporting statistical associations between an SES indicator of at least one parent (education, occupation, income, or an SES index) and different types and intensities of PA in primary school-aged children (6 to 12 years) were included in the analysis. The distributions of the reported associations were evaluated across and differentiated by sub-group analysis of assessment methods (objectively measured vs. self-reported PA) and analysis methods (univariate vs. multivariate models).

**Results:**

Overall, 93 studies reported in 77 publications were included in this review. Most of the studies were conducted in Europe and used self-reports (questionnaires) to assess PA. Most studies used only a single SES indicator (commonly maternal education), and only two studies calculated an SES index. The majority of the studies focused on moderate-to-vigorous physical activity (MVPA), total physical activity (TPA), and organized physical activity (OPA). Results showed predominantly positive associations between SES indicators and OPA. In contrast, results regarding different intensities of daily PA (TPA, LPA, MPA, MVPA, VPA, LTPA) were heterogeneous, with overwhelmingly no associations.

**Conclusion:**

Overall, the results expand the knowledge about the association between family socioeconomic indicators and children’s PA and disprove the hypothesis of a clear positive association. However, large multicenter studies are lacking using a real SES index as a predictor and analyzing gender-specific multivariate models.

**Supplementary Information:**

The online version contains supplementary material available at 10.1186/s12889-024-19174-6.

## Background

There is a large body of evidence demonstrating the relevance of sufficient physical activity (PA) participation for children’s health and development [1–4]. Despite this knowledge, most children worldwide are insufficiently active [5, 6]. Due to the COVID-19 pandemic and concomitant restrictions on out-of-home activities, this problem has reached a new level [7, 8]. Recent findings show that more than 80% of children and adolescents do not reach the recommendation of 60 min of moderate-to-vigorous PA (MVPA) per day [9] and this trend is increasing [10].

Factors influencing PA are diverse and complex. Among others, key determinants influencing PA relate to socioeconomic factors, especially socioeconomic status (SES) [11], usually consisting of education, occupation and (equivalent household) income of parents [12]. This does not only affect short-term health outcomes of children but also lifetime health behavior, including PA participation during adulthood [13–15], which in turn impacts public health in general.

The influence of socioeconomic factors on PA has been investigated in previous systematic reviews for adulthood [16] and for preschool and adolescent age ranges [17]. While results are partially inconsistent, they indicate positive associations for leisure-time PA and a predominantly negative for occupational PA in adults, but no significant associations were identified for children and adolescents. However, there is a lack of systematic evidence syntheses for primary school-aged children (6–12 years). One review examined the relationship between PA and socioeconomic factors during elementary school [18]. However, “payment of fees” (fees parents paid so the child could be active) used as a socioeconomic determinant is only a proxy of socioeconomic status and may not cover non-organized and incidental aspects of PA.

Elementary school age is a sensitive period in terms of PA behavior. With the transition from kindergarten (long periods of free play and time to move) to elementary school (mostly sedentary periods), daily PA decreases significantly [19, 20]. Moreover, the period of youth represents a significant stage of socialization, influencing subsequent behaviors throughout the lifespan, including the formation of a lifelong sufficient PA behavior [21, 22] which is, among other health behaviors, crucial for health outcomes in adulthood [23].

Heterogeneity exists regarding the methodology of available reviews on the associations between PA and socioeconomic status. Often, only one socioeconomic factor is used for analysis (e.g., education). In addition, the PA assessment method has not found sufficient consideration so far. PA assessment in younger age groups (up to 10 years) is not only done mainly by self-report; it also is measured by proxy reports using one or both parents rather than the child itself. This carries the risk of over- or underestimation, aside from other measurement biases such as deliberate changes (e.g., social desirability), item misunderstanding, and misinterpretation [24–26].

In comparison, objective measurement tools (e.g., accelerometer, pedometer) directly collect data from the child and are therefore more precise regarding PA frequency, duration, and intensity. Objective PA assessment, however, has several limitations; specific activities, such as swimming, sliding, or cycling) cannot be recorded or only to a limited extent due to technical limitations [27]. In addition, the measured period is usually brief (e.g., one week) and therefore provides less information about habits and regularity of PA, which is of importance concerning seasonal and weather biases [28]. Due to the various advantages and disadvantages of the different PA measurement methods, it is relvant to consider them separately.

There is clear evidence that boys and girls have different PA amounts and habits [29, 30]. Discrepancies between genders for self-report and objective measures concerning the duration and intensity of PA have also been found [31]. In addition, gender-specific differentiation of parents is an issue that needs to be addressed more. While most studies examine the influence of maternal SES indicators as a benchmark for “parental” SES indicators, paternal SES indicators are less often collected and analyzed. This is probably because mothers are seen as playing a greater and more influential role in the responsibility and organization of childcare [32]. Nevertheless, parents’ roles in developing health-related behaviors vary and affect boys and girls in different ways [33, 34]. Gender-specific analyses of parents and children can also provide more insight regarding potential associations [35].

Taken together, for a better understanding of the impact of socioeconomic indicators and their components on children’s activity behavior, more current and gender-specific research is needed [17, 36], especially for elementary school age, where the influence of family social background is higher than in older age groups [12, 37]. Advanced knowledge of these associations can be used to extend and specify explanatory frameworks. This, in turn, can help improve public health strategy construction. Therefore, the purpose of this article was to: (A) Systematically review the current research about the association between family socioeconomic indicators (education, occupation and household income) and PA among primary school-aged children; (B) Quantify the distribution of reported associations of these distinguished family socioeconomic indicators in children’s PA evaluated by child’s and parents sex; (C) Differentiate this distribution according to assessment methods (objective vs. self-report) and analysis method (univariate vs. multivariate models).

## Subjects and methods

This review was registered with PROSPERO (CRD42021259102) on 15.07.2021. The methodological approach of the review is based on the guidelines of the German Cochrane Community [38]. This manuscript adheres to the PRISMA statement to equity-focused systematic reviews (PRISMA-E) [39].

### Search strategy

Potentially relevant studies from January 2010 until 31st March 2022 were identified by searching five electronic databases (MEDLINE (via PubMed), Web of Science, ScienceDirect, SPORTDiscus, and ERIC). In addition, a hand search was performed using Google Scholar. All databases were searched using combinations of relevant keywords related to exposure and outcome. Database-specific filters about population and language were applied where possible. The specific search strategies for each database are displayed in Table 1.

**Table 1:** Search strategy for databases

Database	Search term	Filter
MEDLINE (via PubMed)	((Socioeconomic inequalities[Text Word]) OR (Socioeconomic inequalit*) OR (Socioeconomic Factors[MeSH Terms]) OR (Social Class[MeSH Terms]) OR (Socioeconomic position[Text Word]) OR (Socioeconomic status[Text Word]) OR (Occupations[MeSH Terms]) OR (Employment[MeSH Terms]) OR (Income[MeSH Terms]) OR (Education[MeSH Terms]) OR (Educational Status[MeSH Terms])) AND ((Sports [MeSH Terms]) OR (Exercise[MeSH Terms]) OR (Physical Activity[Text Word]) OR (Physical Activit*) OR (Physical Fitness[MeSH Terms]) OR (Movement[MeSH Terms])) AND ((Child[MeSH Terms]))	Publication date: 10 Years, Age: Child: 6–12 years, Species: Humans, Language: English
Web of Science	TOPIC: (Socioeconomic inequalitie* OR Socioeconomic Factor* OR Social Class OR Socioeconomic position OR Socioeconomic status OR Occupation* OR Employment OR Income OR Education OR Educational Status) AND TOPIC: (Sport* OR Physical Activity OR Movement) AND TOPIC: (Child*) NOT TOPIC: (Intervention* OR Obesit*)	Language: English, Timespan: 2010–2021
ScienceDirect	Socioeconomic inequalities OR Socioeconomic position OR Socioeconomic status OR Occupation OR Employment OR Income OR Education AND Physical Activity AND Children	Keywords: Physical Activity, Children
SPORTDiscus	Socioeconomic inequalities OR Socioeconomic position OR Socioeconomic status OR Occupation OR Employment OR Income OR Education AND Physical Activity AND Children	Language: English, Publication Type: Articel, Timespam: January 2010 – March 2021
ERIC	Socioeconomic inequalities OR Socioeconomic position OR Socioeconomic status OR Occupation OR Employment OR Income OR Education AND Physical Activity AND Children	Publication date: last 10 years

### Eligibility

Inclusion and exclusion criteria are listed below.

- Population: Children aged between 6 and 12 years.
- Exposure: Index for socioeconomic status (built out of a combination of the three relevant SES indicators. Indicators considered were income (household level) as well as occupation and education of at least one parent or at least one SES indicator.
- Outcome: PA in unorganized (e.g., ‘free play’) or organized (e.g., sports clubs) settings. PA intensities (e.g., total physical activity (TPA)), moderate physical activity (MPA), moderate-to-vigorous physical activity (MVPA), vigorous physical activity (VPA), leisure-time physical activity (LTPA), PA domains (e.g., organized physical activity (OPA)), active transport (AT), PA frequencies (e.g., times/week, steps/day) or PA durations (e.g., hours or minutes). Studies using objective (e.g., accelerometer, pedometer) and self-report (questionnaire) assessment methods were included.
- Study design: Cross-sectional or longitudinal survey studies.
- There was no restriction as to where studies were conducted.

The following exclusion criteria applied:

- Patient groups (any form of physical or mental diseases or disabilities, including overweight or obesity).
- Intervention studies.
- Qualitative study designs.
- Excluded SES indicators: neighborhood SES, household and family wealth (e.g., car or house ownership, housing tenure, family affluence scale (FAS)), area-based indicators (e.g., average country’s income, area deprivation), SES scores constructed not using the three included secioeconomic indicators (income, occupation, education).
- Excluded PA domain (outcome): competitive sport, physical education (PE).

**Fig. 1:**
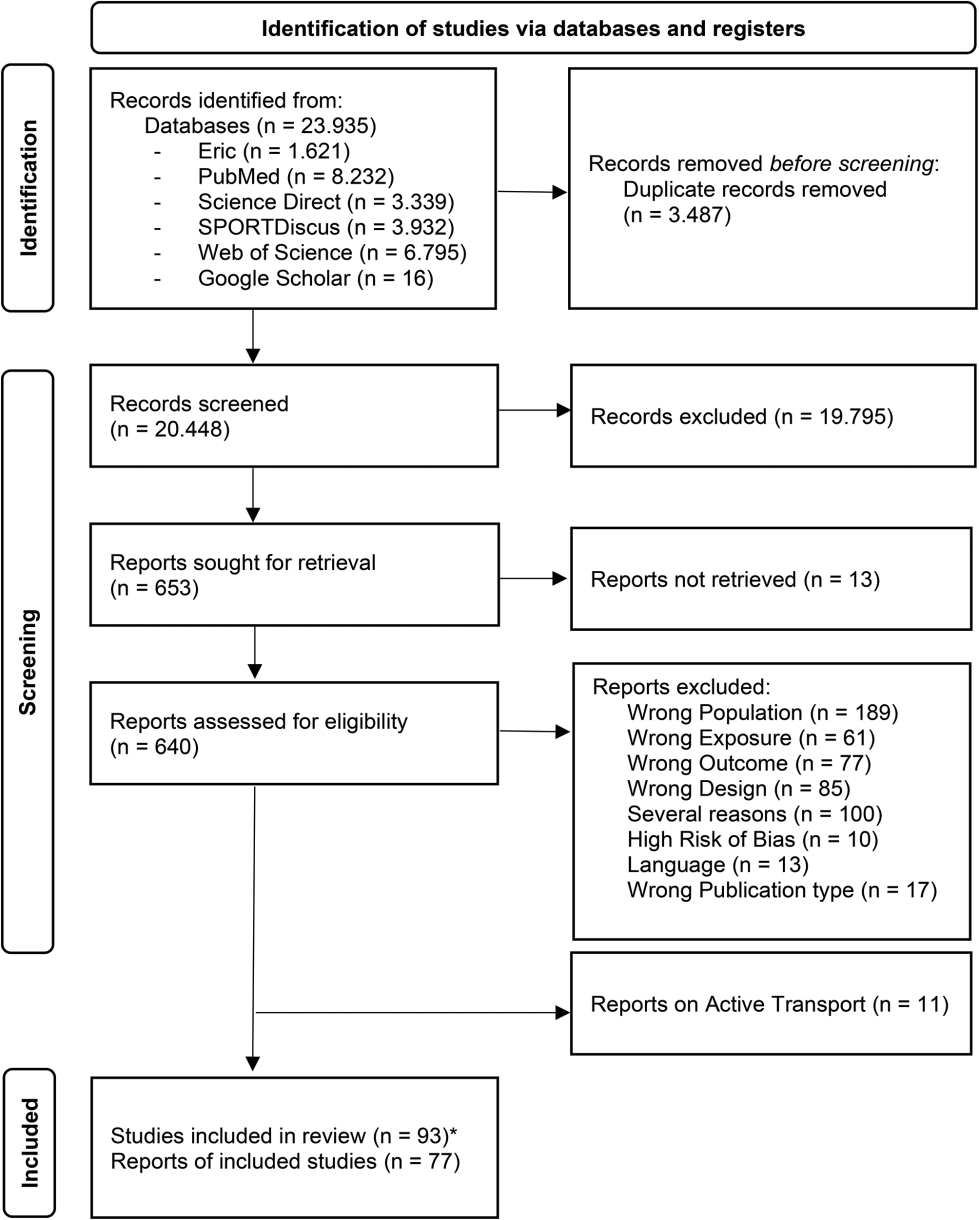
Flow chart diagram. *For better comparison with other single-center studies, multi-country studies were separated into single studies according to country

Although some studies technically fulfilled the inclusion criteria, they had to be excluded for the following reasons: thematic context inappropriate (e.g., analysis refers to group differences between ethnicities or countries), the association between exposure and outcome not reported, qualitative deficiencies (missing data e.g., specific age groups) or a combination for several of these reasons (Fig. 1).

Only articles published in English and peer-reviewed journals were included. Conference abstracts and theses were not considered. In addition, studies were excluded if the risk of bias was considered high (see section risk of bias assessment below).

Due to the amount of data and methodological differences, identified studies for the PA category active transport (AT) will be published separately.

### Selection process

Identified citations from the databases were exported into Endnote, and duplicates were removed following the procedure proposed [40]. Title and abstract screening and eligibility assessment of potentially relevant full-texts were done independently by at least two reviewers (AF, AZ, DS, KB). Disagreements were solved by a discussion involving a third reviewer.

Data extraction was done using a pre-piloted Excel spreadsheet by at least two reviewers. The following information was extracted: author, year, country, period of data collection, sample size, age range, gender percentage, response rate, SES indicators, PA outcomes, and measurement methods (Tables 2 and 3). In studies with age groups or age ranges beyond the defined age range of 6–12 years, only data for this age cohort were extracted (as a result, data reported here may be just partial data and may differ from the data of the total sample from the studies).

**Table 2:** Characteristics of the included studies regarding localisation and year

Author & year of publication	Continent	Country of study	Study name	Year (data)
(Aarts et al., 2012) [71] (a)	Europe	NLD	-	2007–2008
(Aarts et al., 2010) [72] (a)	Europe	NLD	-	2007–2008
(Aggio et al., 2017) [73] (b)	Europe	GBR	Millennium Cohort Study (MCS)	2008–2009 (Actigraph accelerometer)
(Aguilar-Farias et al., 2019) [74]	South America	RCH	ESPACIOS study	NP
(Al Yazeedi et al., 2021) [75]	Asia	OMN	-	2017–2018
(Alotaibi et al., 2020) [76]	Asia	KSA	-	NP
(Atkin et al., 2016) [61] (b)	Europe	GBR	Millennium Cohort Study (MCS)	2008–2010
(Bagordo et al., 2017) [77]	Europe	ITA	MAPEC_LIFE study	2014–2015
(Barr-Anderson et al., 2017) [78] (g)	North America	USA	Transitions and Activity Changes in Kids study (TRACK)	2008–2009 & 2010–2011
(Beckvid Henriksson et al., 2016) [79]	Europe	SWE	-	2010 & 2012
(Brug et al., 2012)* [80] (c)	Europe	*Singel Studies*: BEL, GRC, HUN, NLD, NOR, SVN, ESP	ENERGY-Project	2010
(Butte et al., 2014) [81]	North America	USA	-	2007–2009
(Cadogan et al., 2014) [82]	Europe	IRL	Growing Up in Ireland study	2007–2008
(Cárdenas-Fuentes et al., 2021) [83]	Europe	ESP	POIBC study	2012–2014
(Cvetković et al., 2014) [84]	Europe	SRB	-	NP
(da Silva et al., 2014) [85] (d)	South America	BRA	-	2010–2013
(de Moraes Ferrari et al., 2016) [86] (e)	South America	BRA	ISCOLE Study Brazil	2012–2013
(Deng & Fredriksen, 2018) [87]	Europe	NOR	The Health Oriented Pedagogical Project (HOPP)	2015
(Ding et al., 2020) [88] (d)	South America	BRA	-	2004, 2010–2013
(Dmitruk et al., 2015) [89]	Europe	POL	-	NP
(Drenowatz et al., 2010) [90] (Study 1)	North America	USA	SWITCH	2006
(Drenowatz et al., 2010) [90] (Study 2)	North America	USA	SWITCH	2006 & 2007
(Duncan et al., 2012) [91]	Europe	GBR	-	2011
(Engel-Yeger, 2012) [92]	Asia	ISR	-	NP
(Fakhouri et al., 2013) [93]	North America	USA	NHANES 2009–2010	2009–2010
(Fernández-Alvira et al., 2015)* [94] (c)	Europe	*Singel Studies*: BEL, GRC, HUN, NLD, NOR, SVN, ESP	ENERGY-project	2010
(Gomes et al., 2017) [95] (e)	Europe	PRT	ISCOLE Study Portugal	2011–2013
(Harbec et al., 2021) [96]	North America	CDN	Québec Longitudinal Study of Child Development (QLSCD) birth cohort	2004–2010
(Herzig et al., 2012) [97] (c)	Europe	SWI	ENERGY-project Switzerland	2010
(Huang et al., 2010) [98]	Asia	ROC	-	2004 (measured children’s PA)
(Huang et al., 2013) [99]	Asia	CHN	-	NP
(Janssen et al., 2014) [100]	North America	CDN	First Nations Regional Health Survey	2008–2010
(Jerina et al., 2018) [101]	Europe	SVN	-	2010
(Jiménez-Pavón et al., 2012)* [102] (c)	Europe	*Single Studies*: BEL, GRC, HUN, NLD, NOR, SVN, ESP	ENERGY-project	2010
(Kawalec & Pawlas, 2021) [103]	Europe	POL	-	2017–2019
(Knuth et al., 2017) [104] (d)	South America	BRA	-	2010–2011
(Kobel et al., 2015) [105]	Europe	DEU	-	2010–2011
(Lämmle et al., 2012) [106]	Europe	DEU	Motorik Module study (MoMo)	2003 & 2006
(Lampinen et al., 2017) [46]	Europe	FIN	Physical Activity and Nutrition in Children (PANIC) Study	2007–2009
(Larouche et al., 2019) [107] (e)	Australia, South America, North America, Asia, Africa, Europe	*Cohort Study*: AUS, BR, CDN, CHN, COL, FIN, IND, KEN, PRT, ZAF, GBR, USA	ISCOLE study	2011–2013
(Larouche et al., 2019) [108]	North America	CDN	-	2016–2017
(Lepeleere et al., 2015) [109]	Europe	BEL	-	2014
(Lewis et al., 2016) [110] (e)	Australia	AUS	ISCOLE Australian arm	2011–2012
(Love et al., 2019) [111] (b)	Europe	GBR	Millennium Cohort Study (MCS)	2008–2009
(Manyanga et al., 2019) [112]	Africa	MOC	-	2017–2018
(Manz et al., 2016) [113]	Europe	DEU	KiGGS	2003–2006 (KiGGS0)
(Matsudo et al., 2016) [114] (e)	South America	BRA	ISCOLE Brazil	2012–2013
(McCormack et al., 2011) [115]	Australia	AUS	TRavel, Environment, and Kids project (TREK)	2007
(McMinn et al., 2013) [116]	Europe	GBR	SPEEDY study	2007
(McMinn et al., 2011) [117]	Europe	GBR	Child Heart and Health study (CHASE)	2006–2007
(Moraeus et al., 2012) [118] (f)	Europe	SWE	Childhood Obesity Surveillance Initiative (COSI)	2008
(Moraeus et al., 2015) [119] (f)	Europe	SWE	Childhood Obesity Surveillance Initiative (COSI)	2008, 2010, 2013
(Musić Milanović et al., 2021)* [56]	Europe	*Single Studies*: DNK, IRL, LTU, LVA, BGR, CZE, POL, ROU, FRA, ALB, HRZ, MLT, MNE, PRT, ESP, KAZ, KGZ, TJK, TKM, GEO, TR	Childhood Obesity Surveillance Initiative (COSI)	2015–2017
(Muthuri et al., 2016)* [120] (e)	Australia, South America, North America, Asia, Africa, Europe	*Single Studies*: AUS, BRA, CDN, CHN, COL, FIN, IND, KEN, PRT, ZAF, GBR, USA	ISCOLE Study	2012–2013
(Muthuri et al., 2014) [121] (e)	Africa	KEN	ISCOLE Study	2011–2013
(Nakabazzi et al., 2020) [36]	Africa	UGA	-	2017–2018
(Noonan & Fairclough, 2018) [122] (b)	Europe	GBR	Millennium Cohort Study (MCS)	2001–2002, 2008–2009
(Nyberg et al., 2020) [43]	Europe	SWE	Riksmaten Adolescents 2016-17	2016–2017
(Paduano et al., 2021) [123]	Europe	ITA	-	2018
(Pate et al., 2022) [124] (g)	North America	USA	Transitions and Activity Changes in Kids (TRACK) Study	2010–2017
(Pouliou et al., 2015) [125] (b)	Europe	GBR	Millennium Cohort Study (MCS)	2008–2009
(Rosell et al., 2021) [126]	Europe	SWE	Generation Pep Study	2018
(Sanmarchi et al., 2022) [127]	Europe	ITA	The “Seven Days for My Health” Project	2017
(Schmidt et al., 2022) [128]	Europe	DEU	Motorik Module study (MoMo)	2003–2012, 2020
(Smith et al., 2015) [129]	Europe	GBR	Olympic Regeneration in East London (ORiEL) study	2012
(Tandon et al., 2014)** [130] (h)	North America	USA	Neighborhood Impact on Kids (NIK) Study	2007–2009
(Tandon et al., 2012)** [131] (h)	North America	USA	Neighborhood Impact on Kids (NIK) Study	2007–2009
(Tercedor et al., 2019) [132]	Europe	ESP	PREVIENE project	2017
(To et al., 2020) [133]	Asia	VNM	-	2016
(van Stralen et al., 2014)* [134] (c)	Europe	*Singel Studies*: BEL, GRC, HUN, NLD, SWI	ENERGY-project	2010
(Vandendriessche et al., 2012) [135]	Europe	BEL	-	NP
(Vandermeerschen et al., 2015) [136]	Europe	BEL	-	2009
(Veitch et al., 2010) [137]	Australia	AUS	Children’s Leisure Activities Study Survey (CLASS)	NP
(White & McTeer, 2012) [138]	North America	CDN	National Longitudinal Survey of Children and Youth (NLSCY)	1998–1999
(Wijtzes et al., 2014) [139]	Europe	NLD	Generation R study	2002–2006
(Wilk et al., 2018) [140]	Europe	GBR	Grade 5 ACT-i-Pass (G5AP)	2014–2015
(Wilkie et al., 2018) [141] (e)	Europe	GBR	ISCOLE Study England	2011–2013

ALB = Albania, BEL = Belgium, BGR = Bulgaria, CZE = CzechRepublic, DEU = Germany, DNK = Denmark, ESP = Spain, EST = Estonia, FIN = Finland, FRA = France, GBR = UnitedKingdom, GRC = Greece, GEO = Georgia, HRZ = Croatia (local name is Hrvatska), HUN = Hungary, IRL = Ireland, ITA = Italy, KAZ = Kazakhstan, KGZ = Kyrgyzstan, LTU = Lithuania, LVA = Latvia, MLT = Malta, MNE = Montenegro, NLD = TheNetherlands, NOR = Norway, POL = Poland, PRT = Portugal, ROU = Romania, SRB = Serbia, SVN = Slovenia, SWE = Sweden, SWI = Switzerland, TKM = Turkmenistan, TJK = Tajikistan, AUS = Australia, BRA = Brasil, CDN = Canada, CHN = China, COL = Columbia, IND = India, IRN = Iran, KEN = Kenia, KSA = Saudi Arabia, MOC = Mosambique, MY = Malaysia, OMN = Oman, RCH = Chile, TR = Turkey, UGA = Uganda, USA = United States of America, ROC = Republic of China (Taiwan), VNM = Vietnam, ZAF = South Africa, EU = European Union (more than 5 countries of EU)

NP = Not Provided

* Article includes several studies based on country

** same study cohort -> only Results from Tandon et al. 2012 study included in analyses (Tandon et al. 2014 excluded)

(a) = same study setting, (b) = same study setting (Millennium Cohort Study (MCS)), (c) = same study setting (ENERGY-project), (d) = same study setting, (e) = same study setting (International Study of Childhood Obesity, Lifestyle and the Environment (ISCOLE Study)), (f) = same study setting (Childhood Obesity Surveillance Initiative (COSI)), (g) = same study setting (Transitions and Activity Changes in Kids study (TRACK)), (h) = same study setting (Neighborhood Impact on Kids (NIK) Study), (i) = same study setting (SWITCH)

### Classification

The following guidelines were used to classify PA and SES indicators in this study.

### Outcome measures

PA was categorized in total physical activity (TPA) if no specific information about duration or intensity was provided and/or if described as ‘total,’ ‘usual,’ ‘daily,’ ‘daily steps,’ ‘daily step counts’, or ‘overall’ PA. PA was also categorized based on intensity in vigorous physical activity (VPA), moderate-to-vigorous physical activity (MVPA), moderate physical activity (MPA) or light physical activity (LPA).

Unorganized physical activity was classified as leisure-time physical activity (LTPA) when described as ‘PA in free time’, ‘play time’, ‘free play’ and/or named ‘total Leisure-time physical activity’.

Extracurricular sports, PA in sports clubs, and PA structured/supervised by a coach, instructor, or other leader were categorized as OPA. Extracurricular sport was classified as OPA only if clearly defined as a guided, structured unit. If not, it is categorized as LTPA.

### Socioeconomic indicators

Education (ED) refers to the highest attained level of education (e.g., university education) or the total years of education of one or both parents. Income (IN) refers to the individual income of one (individual level) or both parents (household level). The occupation level (OC) describes the type or amount of employment status of one or both parents. Socioeconomic status (SES) was classified when all three indicators were considered simultaneously in one index.

### Risk of bias assessment

Methods of quality assessment were limited by study type. According to the quality assessment used by Beenackers et al. (2012) [16], quality markers: response rate, adjustment, and sample size were conducted to check if quality aspects affect the study results. Following the full-text screening, all remaining studies were re-assessed using these quality markers. The markers were rated as high risk when the response rate was < 50%, associations were not adjusted for at least one variable (e.g., age, gender, etc.) and if a minimum sample size of 500 was not reached to ensure representativeness. Medium risk was classified when the relevant information was unclear, not provided or unspecified, and low risk was classified when all criteria were met. Studies were excluded if two or more quality markers were rated as high risk or if one quality marker was rated as high risk and the other two as medium risk. The risk of bias assessment for the included studies is shown in an additional file (see Additional file 1).

### Synthesis of results

Due to high levels of heterogeneity related to population, exposure and outcomes, we refrained from conducting meta-analysis. Studies conducted separately in multiple countries and published as one trial were separated to enhance comparability with other studies. Similar to previous syntheses [16, 40, 41], the results of studies were analyzed on the level of the separate associations instead of just analyzing the complete studies to facilitate understanding of the different associations between various PA domains and various SES indicators.

The associations between the domains of PA behavior and the SES indicators were labeled with ‘+’ when the association was positive and significant. The association was tagged with ‘0’ when no significant association existed. The association was tagged with ‘-’ when a significant negative association was found. A significance level α = 0.05 was considered. In the case of more than two groups, comparisons between them were included based on the applied statistical methods in the original studies.

If available, the adjusted results were used to account for confounding factors. Distributions of reported positive, negative, and null associations were evaluated for each PA outcome by gender and SES indicator (Tables 4, 5, 6, 7 and 8). The same analyses were conducted for both genders, combined as well as separated. Sub-group analyses were performed for self-report versus objective PA assessments and for univariate versus multivariate analyses.

**Table 3:** Characteristics of the included studies regarding sample, SES and PA

Author & year of publication	Sample (*n*)	Age in years (mean) or grade	% Male	Response Rate %	SES indicator	PA, Measurement method
(Aarts et al., 2012) [71] (a)	1.357 (7–9 years), 1.046 (10–12 years)	7–9 & 10–12	51% (7–9 years) 49.7% (10–12 years)	60% (only provided for total sample)	Education	LTPA (outdoor play, min/week), subjective (Questionnaire)
(Aarts et al., 2010) [72] (a)	2.383 (7–9 years), 1.914 (10–12 years)	7–9 & 10–12	51.9% (7–9 years) 49% (10–12 years)	60% (only provided for total sample)	Education	LTPA (outdoor play, min/week), subjective (Questionnaire)
(Aggio et al., 2017) [73] (b)	6.442 (accelerometer sample), 13.169 (Survey sample)	7	48.9% (Accelero-meter sample) 50.7% (Survey sample)	NP	Income	LTPA, MVPA, objective (waist worn accelerometry)
(Aguilar-Farias et al., 2019) [74]	148	9–11 (10.0 ± 0.82)	47.3%	NP *(57.4% (148 of 258 participants included in the final analysis))*	Education, Income	LPA, MPA, VPA, MVPA, objective (ActiGraph GT3X accelerometer
(Al Yazeedi et al., 2021) [75]	197 (dyads)	7.74 ± 1.16	47.4%	NP *(96.6% (197 of 204 enrolled dyads were included))*	Education, Occupation, Income	MVPA, subjective (Child nutrition and PA questionnaire (FFS))
(Alotaibi et al., 2020) [76]	458	6–12 (8.44 ± 2.07)	53.3%	NP *(52 responses were excluded because of incomplete questionnaires, 458 parents responded)*	Education, Income	TPA, subjective (parent-reported survey, C-PAQ)
(Atkin et al., 2016) [61] (b)	704	7.6 (± 0.3)	47.4%	55%	Income	MVPA, Objective (ActiGraph accelerometer (GT1M))
(Bagordo et al., 2017) [77]	1.164	6–8 (7.34 ± 0.87)	50.9%	56.2%	Education, Occupation	LTPA, subjective
(Barr-Anderson et al., 2017) [78] (g)	643	5th -7th grade	45.9%	60% (recruitment rate − 5th grade); 85% (retention rate − 7th grade)	Education	TPA, objective (ActiGraph triaxial accelerometer (GT1M and GT3X))
(Beckvid Henriksson et al., 2016) [79]	621	6.3 (± 0.32)	50.3%	76% (2010), 57% (2012)	Education	MVPA, VPA, TPA, objective (ActiGraph triaxial accelerometer (GT3X+)
(Brug et al., 2012)* [80] (c)	*7.234 (total sample)*, 666 (BEL), 891 (GRC), 763 (HUN), 349 (NLD), 716 (NOR), 895 (SVN), 879 (ESP)	10–12 (11.6 ± 0.7)	48%	> 80%; (exeptions: HUN, NOR, ESP)	Education	OPA, subjective
(Butte et al., 2014) [81]	282	8–10	47.2%	NP	Education, Income	MVPA, objective (actical accelerometer-based monitors)
(Cadogan et al., 2014) [82]	8.568	9	48.6%	82.3% (school level), 57% (household level)	Education, Occupation	MVPA, subjective
(Cárdenas-Fuentes et al., 2021) [83]	1.405	8–10 (10.1 ± 0.6)	50.3%	NP	Education	TPA, subjective (physical activity questionnaire for children (PAQ-C))
(Cvetković et al., 2014) [84]	1.630	1st -4th grade	49%	NP	Income, Education	OPA, subjective
(da Silva et al., 2014) [85] (d)	2.636 (valid accelerometry data from Follow-Up at the age of 6 years of all newborns in 2004 (birth cohort study))	6.7 (± 0.19)	51.5%	NP *(69.1% of the eligible participants from the 2004 cohort were included)*	Education	TPA, MVPA, objective (GENEActiv accelerometer)
(de Moraes Ferrari et al., 2016) [86] (e)	328	9–11	51.5%	NP	Education, Income	MVPA, objective (ActiGraph GT3X+)
(Deng & Fredriksen, 2018) [87]	2.123	6–12	50.4%	75.4%	Education	MVPA, objective (Accelerometer)
(Ding et al., 2020) [88] (d)	2.603 (2004 cohort)	6.7 (± 0.3) (2004 cohort)	51.5%	62% (of baseline participants)	Education, Income	MVPA, objective (GENEActive accelerometer)
(Dmitruk et al., 2015) [89]	404	10–12	61%	65%	Education, Occupation, Income	LTPA, subjective
(Drenowatz et al., 2010) [90] (Study 1)	271 (Caucasian (88.0%), African American (3.3%), Hispanic (1.5%), and other (7.3%))	8–11 (9.6 ± 0.9)	43.2%	65%	Income	TPA (steps per day), objective (pedometer (Digiwalker 200-SW))
(Drenowatz et al., 2010) [90] (Study 2)	131 (children; 93.7% Caucasian)	8–11 (7.8 ± 2.3)	48.1%	NP	Income	MVPA, objective (accelermoter (Actigraph GT1M)
(Duncan et al., 2012) [91]	536	8–11 (9.6 ± 1.0)	47.6%	NP	Income	TPA (steps per day), objective (pedometer (NL-2000))
(Engel-Yeger, 2012) [92]	90	6–10,6	65.6%	NP	Education	TPA, subjective
(Fakhouri et al., 2013) [93]	1.218	6–11	50.9%	NP	Income, Education	TPA, subjective
(Fernández-Alvira et al., 2015)* [94] (c)	*5.729 (total sample)*, 624 (BEL), 839 (GRC), 742 (HUN), 309 (NLD), 664 (NOR), 836 (SVN), 835 (ESP)	10–12	46.8%	NP	Education	OPA, subjective
(Gomes et al., 2017) [95] (e)	499	9–11	43.1%	95.7%	Income	MVPA, Objective (Accelerometer (Actigraph GT3X+))
(Harbec et al., 2021) [96]	966 (complete data on classroom engagement at age 6 years)	6.1 (± 0.3)	46.8%	NP	Education, Income	OPA, LTPA, subjective
(Herzig et al., 2012) [97] (c)	546 (Switzerland); 7.148 (EU)	10–12 (11.6 ±0.8 Switzerland) 10–12 (11,5 ± 0,8 EU)	52%	49.5%	Education	OPA, subjective
(Huang et al., 2010) [98]	523 (*n* = 200 urban schools, *n* = 323 rural schools)	11–12	52% (urban schools) 47.1% (rural schools)	72%	Income, Education	TPA, subjective (questionnaire (CAAL and IPAQ))
(Huang et al., 2013) [99]	303 (sub-sample)	11.2 (boys), 11.1 (girls)	47.2%	NP (sub-sempel) (52.8% total sample)	Education	MVPA, subjective (children’s Leisure Activities Study Survey questionnaire-Chinese version (CLASS-C)
(Janssen et al., 2014) [100]	3.184 (*n* = 1.550 -> 6–8 years (47.5%) + *n* = 1.634 -> 9–11 years (52.5%)	6–8 & 9–11	49.2%	72.5%	Education	MVPA, subjective
(Jerina et al., 2018) [101]	669	9–11 (9.9 ± 0.8)	48.8%	NP	Income	TPA, subjective
(Jiménez-Pavón et al., 2012)* [102] (c)	*7.214 (total sample ◊ different data information’s in text (n = 7214) and in Table 1* (*n* = 7213)) 988 (BEL), 1.087 (GRC), 1.028 (HUN), 919 (NLD), 996 (NOR), 1.174 (SVN), 1.021 (ESP)	10–12	47.7%	NP	Education	TPA, subjective
(Kawalec & Pawlas, 2021) [103]	223	7–10 (8.7 ± 0.5)	44.4%	NP	Education, Occupation	LTPA, subjective
(Knuth et al., 2017) [104] (d)	2.604 (valid accelerometry data from Follow-Up at the age of 6 years of all newborns in 2004 (birth cohort study))	6	51.5%	NP *(61.5%) (n* = 2.604 -> 6-year-follow-up cohort vs. *n* = 4.231 -> original birth cohort at 2004)	Education	TPA, Objective (GENEActiv accelerometer model)
(Kobel et al., 2015) [105]	1.714	7.1 (± 0.6)	50%	NP	Education, Income	MVPA, OPA, subjective
(Lämmle et al., 2012) [106]	870	6–9 (7.72 ± 1.04)	50%	66,6%	SES (Mean Score of occupation, income, education)	TPA, subjective
(Lampinen et al., 2017) [46]	486	6–8 ((7.6 (± 0.4) girl)/(7.7 (± 0.4) boys))	51%	NP	Education, Income	TPA, LTPA, OPA, subjective (Questionnaire (PANIC))
(Larouche et al., 2019) [107] (e)	6.478 (total sample)	9–11	45.6%	56.6%	Education	MVPA, LPA, objective
(Larouche et al., 2019) [108]	1.699	8–12	45%	54.2%	Occupation, Education	MVPA, TPA, objective
(Lepeleere et al., 2015) [109]	207 (parent-child pairs)	6–12 (9.4 ± 1.6)	51.7%	87%	Education	TPA, subjective (Questionnaire (FPAQ))
(Lewis et al., 2016) [110] (e)	528	9–11 (10,8 ± 0.5)	45.8%	57% (Response rate of schools 43%)	Income	MVPA, objective (Actigraph GT3X+)
(Love et al., 2019) [111] (b)	5.172	7–8	49.8%	NP	Education, Income	VPA, MPA, objective (Actigraph GT1M)
(Manyanga et al., 2019) [112]	683	9–11 (10.1 ± 0.8)	47.1%	NP	Education, Occupation	MVPA, objective (Actigraph GT3X+)
(Manz et al., 2016) [113]	3.471 (KiGGS0)	6–10 (8.5) (KiGGS0)	50.9%	66.6% (KiGGS0)	Education, Income	OPA, subjective (Questionnaire)
(Matsudo et al., 2016) [114] (e)	485	9–11 years	49%	NP	Income, Occupation, Education	MVPA, objective (Actigraph GT3X+)
(McCormack et al., 2011) [115]	927	10–12	45.7%	56.6%	Education	TPA, subjective
(McMinn et al., 2013) [116]	1.608	9–10 (10.3 ± 0.3)	44.7%	57%	Education	MVPA, objective (ActiGraph GT1M)
(McMinn et al., 2011) [117]	2.071 (valid data)	9–10 (9.95 ± 0.38)	47.8%	69%	Occupation	TPA, objective (ActiGraph GT1M)
(Moraeus et al., 2012) [118] (f)	3.636	7–9	51.6%	80%	Education	LTPA, OPA, subjective (Questionnaire)
(Moraeus et al., 2015) [119] (f)	833 (2008), 1.085 (2010), 1.135 (2013)	7–9	53% (2008), 49% (2010), 51% (2013)	82%	Education	OPA, subjective (Questionnaire)
(Musić Milanović et al., 2021)* [56]	*124.700 (total sample)*, 878 (DNK), 802 (IRL), 3.436 (LTU), 5.071 (LVA), 3.217 (BGR), 1.342 (CZE), 2.646 (POL), 5.736 (ROU), 4.462 (FRA), 2.184 (ALB), 2.520 (HRZ), 2.813 (MLT), 2.613 (MNE), 5.458 (PRT), 9.755 (ESP), 3.598 (KAZ), 5.790 (KGZ), 2.924 (TJK), 3.518 (TKM), 2.950 (GEO), 10.190 (TR)	6–9	51.4%	NP	Education, Occupation	OPA, VPA, subjective (Questionnaire)
(Muthuri et al., 2016)* [120] (e)	*4.752 (total sample)*, 377 (AUS), 342 (BRA), 464 (CDN), 464 (CHN), 573 (COL), 425 (FIN), 460 (IND), 303 (KEN), 537 (PRT), 134 (ZAF), 328 (GBR), 345 (USA)	9–11	48,5% (AUS), 49.1% (BRA), 41.2% (CAN), 53.4% (CHN), 51.5% (COL), 47.8% (FIN), 45.2% (IND), 46.2% (KEN), 44% (PRT), 48.5% (ZAF), 43.9% (GBR), 38% (USA)	64.5% (around)	Education	MVPA, objective (ActiGraph GT3X+)
(Muthuri et al., 2014) [121] (e)	563	9–11	46.5%	44.1%	Education, Income	MVPA, objective (ActiGraph GT3X+)
(Nakabazzi et al., 2020) [36]	256	10–12	44.1%	42.7%	Education	MVPA, objective (ActiGraph GT3X+)
(Noonan & Fairclough, 2018) [122] (b)	3.717	7	49.2%	NP	Education	MVPA, objective (ActiGraph GT1M)
(Nyberg et al., 2020) [43]	1.217 (total)	11–12 (11.5 ± 0.4)	48.9%	NP	Education	TPA, LPA, MPA, VPA, MVPA, objective (ActiGraph GT3X & GT3X+)
(Paduano et al., 2021) [123]	558	6–7	53.2%	74.2% (of total sample)	Education	OPA, subjective (Questionnaire)
(Pate et al., 2022) [124] (g)	951 (5th grade children who were measured at least once over the 5 data collection periods)	10.6 (± 0.5)	44.6%	NP	Education	TPA, objective (ActiGraph accelerometers (GT1M & GT3X))
(Pouliou et al., 2015) [125] (b)	6.497	7	50.9%	72%	Education	TPA, MVPA, objective (Actigraph GT1M)
(Rosell et al., 2021) [126]	12.441 (of total sample ◊ NP for sub-sample 7–12 years)	7–12 (sub- sample)	NP	43% (of total sample)	Education	TPA, subjective (Questionnaire)
(Sanmarchi et al., 2022) [127]	368	8.95 (± 1.43)	46.5%	NP	Education, Occupation	MVPA, subjective (Questionnaire)
(Schmidt et al., 2022) [128]	647	6–10 (8.3 ± 1.4)	53.3%	NP	SES Index (Education, Occupation, Income)	OPA, MVPA, subjective (Questionnaire (MoMo-PAQ))
(Smith et al., 2015) [129]	3.105	11–12	56.6%	NP	Occupation	TPA, subjective (Questionnaire (Y-PAQ))
(Tandon et al., 2014)** [130] (h)	713	6–11 (9.2 ± 1.6)	51%	NP	Income	MVPA, objective (Actigraph GT1M)
(Tandon et al., 2012)** [131] (h)	713 (children-parent pairs)	6–11	NP	NP	Education; Income	MVPA, objective (Actigraph GT1M)
(Tercedor et al., 2019) [132]	291	8.3 (± 0.3)	53.6%	NP	Education	LPA, MPA, VPA, MVPA, objective (Actigraph wGT3X-BT)
(To et al., 2020) [133]	619	125.0 (± 2.3) months (10.42 years)	61.3%	61.3%	Income, Education	TPA, objective (Digiwalker SW200)
(van Stralen et al., 2014)* [134] (c)	*1.025 (total sample)*, 190 (BEL), 201 (GRC), 178 (HUN), 190 (NLD), 265 (SWI)	10–12 (11.6 ± 0.9)	49%	NP	Education	MVPA, objective (ActiTrainers, GT3X, GT1M)
(Vandendriessche et al., 2012) [135]	1.955	6–7, 8–9, 10–11	52%	NP	Occupation	LTPA, subjective (Questionnaire)
(Vandermeerschen et al., 2015) [136]	2.103	6–12 (sub-sample)	50.6% (total sample)	68% (total sample)	Income, Education	OPA, subjective (Questionnaire)
(Veitch et al., 2010) [137]	187	8–9 (9.1 ± 0.4)	53%	NP	Education	LTPA, subjective & objective (survey & accelerometer (Actigraph 7164))
(White & McTeer, 2012) [138]	4.412 (unorganized PA), 4.413 (organized PA)	6–9	50.6%	NP	Education, Occupation, Income	LTPA, OPA, Subjective (Questionnaire)
(Wijtzes et al., 2014) [139]	4.685 (OPA), 3.903 (LTPA)	6 (73.0 ± 5.9 months)	50.5%	NP	Education, Income, Occupation	LTPA, OPA, subjective (Questionnaire)
(Wilk et al., 2018) [140]	1.517	9–11	50.1%	84.3% (parents), 56% (children)	Education, Occupation, Income	TPA, subjective (Physical Activity Questionnaire for Children (PAQ-C))
(Wilkie et al., 2018) [141] (e)	462	9–11 (10.9 ± 0.5)	45%	NP	Education	LTPA, Subjective(Questionnaire)

TPA = Total Physical Activity, LPA = Light Physical Activity, MPA = Moderate Physical Activity, VPA = Vigorous Physical Activity, MVPA = Moderate-to-vigorous Physical Activity, LTPA = Leisure-time Physical Activity, OPA = Organized Physical Activity

NP = Not Provided

* Article includes several studies based on country

** same study cohort -> only Results from Tandon et al. 2012 study included in analyses (Tandon et al. 2014 excluded)

(a) = same study setting, (b) = same study setting (Millennium Cohort Study (MCS)), (c) = same study setting (ENERGY-project), (d) = same study setting, (e) = same study setting (International Study of Childhood Obesity, Lifestyle and the Environment (ISCOLE Study)), (f) = same study setting (Childhood Obesity Surveillance Initiative (COSI)), (g) = same study setting (Transitions and Activity Changes in Kids study (TRACK)), (h) = same study setting (Neighborhood Impact on Kids (NIK) Study), (i) = same study setting (SWITCH)

**Table 4:** Distribution of positive, null and negative associations by PA domain, sex and SES indicator

		Parental education				Maternal education				Paternal education				Household income				Parental occupation				Maternal occupation				Paternal occupation				SES			
PA			+	0	-		+	0	-		+	0	-		+	0	-		+	0	-		+	0	-		+	0	-		+	0	-
	*sex*	N	%	%	%	N	%	%	%	N	%	%	%	N	%	%	%	N	%	%	%	N	%	%	%	N	%	%	%	N	%	%	%
**TPA**	*m*	4	25	75	0	9	33	56	11	8	50	50	0	3	0	100	0	1	0	100	0	1	0	100	0	0				0			
	*f*	5	20	80	0	9	22	67	11	8	12,5	87,5	0	3	33	67	0	1	0	100	0	1	0	100	0	0				0			
	*all*	9	0	67	33	6	0	50	50	2	0	50	50	9	22	67	11	2	0	100	0	3	0	100	0	2	0	100	0	1	100	0	0
**VPA**	*m*	1	0	100	0	0				0				0				0				0				0				0			
	*f*	1	100	0	0	0				0				0				0				0				0				0			
	*all*	22	13,5	54,5	32	3	33	67	0	1	0	100	0	2	50	50	0	18	0	83	17	1	0	100	0	0				0			
**MVPA**	*m*	5	0	80	20	2	0	50	50	1	0	100	0	2	0	50	50	1	0	100	0	2	0	50	50	1	0	100	0	1	0	100	0
	*f*	5	20	40	40	2	0	50	50	1	100	0	0	2	0	50	50	1	0	100	0	2	0	100	0	1	0	100	0	1	0	100	0
	*all*	13	0	85	15	20	10	45	45	16	6	75	19	12	8,3	58,3	33,3	0				5	0	80	20	3	0	67	33	0			
**MPA**	*m*	1	0	100	0	0				0				0				0				0				0				0			
	*f*	1	0	100	0	0				0				0				0				0				0				0			
	*all*	2	0	100	0	2	0	50	50	1	0	100	0	2	0	50	50	0				1	0	100	0	0				0			
**LPA**	*m*	2	0	50	50	0				0				0				0				0				0				0			
	*f*	2	0	50	50	0				0				0				0				0				0				0			
	*all*	2	0	100	0	1	0	100	0	1	0	100	0	1	0	0	100	0				0				0				0			
**LTPA**	*m*	2	0	50	50	2	0	100	0	1	0	100	0	3	33	67	0	1	100	0	0	1	0	100	0	1	0	100	0	0			
	*f*	2	0	50	50	2	0	100	0	1	0	100	0	3	0	100	0	1	100	0	0	1	0	100	0	1	0	100	0	0			
	*all*	5	40	20	40	3	0	33	67	3	0	33	67	3	33,3	33,3	33,3	2	0	100	0	2	0	100	0	3	33,3	33,3	33,3	1	0	100	0
**OPA**	*m*	1	100	0	0	1	0	0	100	0				2	50	0	50	0				0				0				0			
	*f*	1	100	0	0	1	0	0	100	0				2	50	0	50	0				0				0				0			
	*all*	30	80	20	0	5	80	20	0	5	80	20	0	6	83	17	0	19	68	32	0	1	0	100	0	1	0	100	0	0			

+ = significant positive association, 0 = no significant (linear), - = significant negative association, m = male, f = female, all = male and female combined (no gender separation)

**Table 5:** Sub-group analysis by PA objective assessment method for distribution of positive, null and negative associations by PA domain, sex and SES indicator

Obj		Parental education				Maternal education				Paternal education				Household income				Parental occupation				Maternal occupation				Paternal occupation				SES			
PA			+	0	-		+	0	-		+	0	-		+	0	-		+	0	-		+	0	-		+	0	-		+	0	-
	*sex*	N	%	%	%	N	%	%	%	N	%	%	%	N	%	%	%	N	%	%	%	N	%	%	%	N	%	%	%	N	%	%	%
**TPA**	*m*	2	0	100	0	1	0	0	100	0				1	0	100	0	1	0	100	0	1	0	100	0	0				0			
	*f*	2	50	50	0	1	0	0	100	0				1	0	100	0	1	0	100	0	1	0	100	0	0				0			
	*all*	5	0	80	20	2	0	50	50	1	0	100	00	5	40	60	0	1	0	100	0	2	0	100	0	1	0	100	0	0			
**VPA**	*m*	1	0	100	0	0				0				0				0				0				0				0			
	*f*	1	100	0	0	0				0				0				0				0				0				0			
	*all*	3	0	100	0	2	50	50	0	1	0	100	0	2	50	50	0	0				0				0				0			
**MVPA**	*m*	4	0	75	25	2	0	50	50	1	0	100	0	2	0	50	50	1	0	100	0	2	0	50	50	1	0	100	0	0			
	*f*	4	25	25	50	2	0	50	50	1	100	0	0	2	0	50	50	1	0	100	0	2	0	100	0	1	0	100	0	0			
	*all*	7	0	71	29	17	0	53	47	14	0	86	14	10	10	50	40	0				3	0	67	33	2	0	100	0	0			
**MPA**	*m*	1	0	100	0	0				0				0				0				0				0				0			
	*f*	1	0	100	0	0				0				0				0				0				0				0			
	*all*	2	0	100	0	2	0	50	50	1	0	100	0	2	0	50	50	0				0				0				0			
**LPA**	*m*	2	0	50	50	0				0				0				0				0				0				0			
	*f*	2	0	50	50	0				0				0				0				0				0				0			
	*all*	2	0	100	0	1	0	100	0	1	0	100	0	1	0	0	100	0				0				0				0			
**LTPA**	*m*	0				0				0				0				0				0				0				0			
	*f*	0				0				0				0				0				0				0				0			
	*all*	0				0				0				0				0				0				0				0			
**OPA**	*m*	0				0				0				0				0				0				0				0			
	*f*	0				0				0				0				0				0				0				0			
	*all*	0				0				0				0				0				0				0				0			

+ = significant positive association, 0 = no significant (linear), - = significant negative association, m = male, f = female, all = male and female combined (no gender separation), obj = objectiv

**Table 6:** Sub-group analysis by PA self-report assessment method for distribution of positive, null and negative associations by PA domain, sex and SES indicator

SR		Parental education				Maternal education				Paternal education				Household income				Parental occupation				Maternal occupation				Paternal occupation				SES			
PA			+	0	-		+	0	-		+	0	-		+	0	-		+	0	-		+	0	-		+	0	-		+	0	-
	*sex*	N	%	%	%	N	%	%	%	N	%	%	%	N	%	%	%	N	%	%	%	N	%	%	%	N	%	%	%	N	%	%	%
**TPA**	*m*	2	50	50	0	8	37,5	62,5	0	8	50	50	0	2	0	100	0	0				0				0				0			
	*f*	3	0	100	0	8	25	75	0	8	12,5	87,5	0	2	50	50	0	0				0				0				0			
	*all*	4	0	50	50	4	0	50	50	1	0	0	100	4	0	75	25	1	0	100	0	1	0	100	0	1	0	100	0	1	100	0	0
**VPA**	*m*	0				0				0				0				0				0				0				0			
	*f*	0				0				0				0				0				0				0				0			
	*all*	19	16	47	37	1	0	100	0	0				0				18	0	83	17	1	0	100	0	0				0			
**MVPA**	*m*	1	0	100	0	0				0				0				0				0				0				1	0	100	0
	*f*	1	0	100	0	0				0				0				0				0				0				1	0	100	0
	*all*	6	0	100	0	3	67	0	33	2	50	0	50	2	0	100	00	0				2	0	100	0	1	0	0	100	0			
**MPA**	*m*	0				0				0				0				0				0				0				0			
	*f*	0				0				0				0				0				0				0				0			
	*all*	0				0				0				0				0				1	0	100	0	0				0			
**LPA**	*m*	0				0				0				0				0				0				0				0			
	*f*	0				0				0				0				0				0				0				0			
	*all*	0				0				0				0				0				0				0				0			
**LTPA**	*m*	2	0	50	50	2	0	100	0	1	0	100	0	3	33	67	0	1	100	0	0	1	0	100	0	1	0	100	0	0			
	*f*	2	0	50	50	2	0	100	0	1	0	100	0	3	0	100	0	1	100	0	0	1	0	100	0	1	0	100	0	0			
	*all*	5	40	20	40	3	0	33	67	3	0	33	67	3	33,3	33,3	33,3	2	0	100	0	2	0	100	0	3	33,3	33,3	33,3	1	0	100	0
**OPA**	*m*	1	100	0	0	1	0	0	100	0				2	50	0	50	0				0				0				0			
	*f*	1	100	0	0	1	0	0	100	0				2	50	0	50	0				0				0				0			
	*all*	30	80	20	0	5	80	20	0	5	80	20	0	6	83	17	0	19	68	32	0	1	0	100	0	1	0	100	0	0			

+ = significant positive association, 0 = no significant (linear), - = significant negative association, m = male, f = female, all = male and female combined (no gender separation), sr = self-report

**Table 7:** Sub-group analysis by multivariate analysis method for distribution of positive, null and negative associations by PA domain, sex and SES indicator

MV		Parental education				Maternal education				Paternal education				Household income				Parental occupation				Maternal occupation				Paternal occupation				SES			
PA			+	0	-		+	0	-		+	0	-		+	0	-		+	0	-		+	0	-		+	0	-		+	0	-
	*sex*	N	%	%	%	N	%	%	%	N	%	%	%	N	%	%	%	N	%	%	%	N	%	%	%	N	%	%	%	N	%	%	%
**TPA**	*m*	2	0	100	0	8	25	62,5	12,5	7	57	43	0	2	0	100	0	1	0	100	0	1	0	100	0	0				0			
	*f*	3	0	100	0	8	12,5	75	12,5	7	14	86	0	2	0	100	0	1	0	100	0	1	0	100	0	0				0			
	*all*	7	0	71	29	5	0	60	40	2	0	50	50	8	25	62,5	12,5	2	0	100	0	3	0	100	0	2	0	100	0	1	100	0	0
**VPA**	*m*	0				0				0				0				0				0				0				0			
	*f*	0				0				0				0				0				0				0				0			
	*all*	21	14,3	52,4	33,3	3	33	67	0	1	0	100	0	2	50	50	0	18	0	83	17	1	0	100	0	0				0			
**MVPA**	*m*	3	0	67	33	2	0	50	50	1	0	100	0	2	0	50	50	1	0	100	0	2	0	50	50	1	0	100	0	0			
	*f*	3	0	67	33	2	0	50	50	1	100	0	0	2	0	50	50	1	0	100	0	2	0	100	0	1	0	100	0	0			
	*all*	12	0	83	17	19	5	47,5	47,5	15	0	80	20	10	0	60	40	0				5	0	80	20	3	0	67	33	0			
**MPA**	*m*	0				0				0				0				0				0				0				0			
	*f*	0				0				0				0				0				0				0				0			
	*all*	1	0	100	0	2	0	50	50	1	0	100	0	2	0	50	50	0				1	0	100	0	0				0			
**LPA**	*m*	1	0	0	100	0				0				0				0				0				0				0			
	*f*	1	0	0	100	0				0				0				0				0				0				0			
	*all*	1	0	100	0	1	0	100	0	1	0	100	0	1	0	0	100	0				0				0				0			
**LTPA**	*m*	2	0	50	50	0				0				1	0	100	0	1	100	0	0	0				0				0			
	*f*	2	0	50	50	0				0				1	0	100	0	1	100	0	0	0				0				0			
	*all*	4	50	25	25	3	0	33	67	3	0	33	67	3	33,3	33,3	33,3	2	0	100	0	2	0	100	0	2	50	50	0	0			
**OPA**	*m*	1	100	0	0	0				0				1	100	0	0	0				0				0				0			
	*f*	1	100	0	0	0				0				1	100	0	0	0				0				0				0			
	*all*	29	83	17	0	2	100	0	0	2	100	0	0	4	100	0	0	19	68	32	0	1	0	100	0	1	0	100	0	0			

+ = significant positive association, 0 = no significant (linear), - = significant negative association, m = male, f = female, all = male and female combined (no gender separation), mv = multivariate analysis

**Table 8:** Sub-group analysis by univariate analysis method for distribution of positive, null and negative associations by PA domain, sex and SES indicator

uv		parental education				maternal education				paternal education				household income				parental occupation				maternal occupation				paternal occupation				SES			
PA			+	0	-		+	0	-		+	0	-		+	0	-		+	0	-		+	0	-		+	0	-		+	0	-
	*sex*	N	%	%	%	N	%	%	%	N	%	%	%	N	%	%	%	N	%	%	%	N	%	%	%	N	%	%	%	N	%	%	%
**TPA**	*m*	2	50	50	0	1	100	0	0	1	0	100	0	1	0	100	0	0				0				0				0			
	*f*	2	50	50	0	1	100	0	0	1	0	100	0	1	100	0	0	0				0				0				0			
	*all*	2	0	50	50	1	0	0	100	0				1	0	100	0	0				0				0				0			
**VPA**	*m*	1	0	100	0	0				0				0				0				0				0				0			
	*f*	1	100	0	0	0				0				0				0				0				0				0			
	*all*	1	0	100	0	0				0				0				0				0				0				0			
**MVPA**	*m*	2	0	100	0	0				0				0				0				0				0				1	0	100	0
	*f*	2	50	0	50	0				0				0				0				0				0				1	0	100	0
	*all*	1	0	100	0	1	100	0	0	1	100	0	0	2	50	50	0	0				0				0				0			
**MPA**	*m*	1	0	100	0	0				0				0				0				0				0				0			
	*f*	1	0	100	0	0				0				0				0				0				0				0			
	*all*	1	0	100	0	0				0				0				0				0				0				0			
**LPA**	*m*	1	0	100	0	0				0				0				0				0				0				0			
	*f*	1	0	100	0	0				0				0				0				0				0				0			
	*all*	1	0	100	0	0				0				0				0				0				0				0			
**LTPA**	*m*	0				2	0	100	0	1	0	100	0	2	50	50	0	0				1	0	100	0	1	0	100	0	0			
	*f*	0				2	0	100	0	1	0	100	0	2	0	100	0	0				1	0	100	0	1	0	100	0	0			
	*all*	1	0	0	100	0				0				0				0				0				1	0	0	100	1	0	100	0
**OPA**	*m*	0				1	0	0	100	0				1	0	0	100	0				0				0				0			
	*f*	0				1	0	0	100	0				1	0	0	100	0				0				0				0			
	*all*	1	0	100	0	3	67	33	0	3	67	33	0	2	50	50	00	0				0				0				0			

+ = significant positive association, 0 = no significant (linear), - = significant negative association, m = male, f = female, all = male and female combined (no gender separation), uv = univariate analysis

For better comparison with other single-center studies, multi-country studies were separated into single studies according to country (i.e., publications from EuropeaN Energy balance Research to prevent excessive weight Gain among Youth (ENERGY-project), etc.). Respective studies are marked in Tables 2 and 3.

### Ethics

Approval by an ethics committee was not required as only published data were used in this systematic review.

## Results

The initial yielded a total of 23.935 citations, of which 653 were retrieved as full-text after duplicates removal and title and abstract screening. Finally, 77 reports of 93 studies fulfilled the eligibility criteria and were included. The entire study selection process is displayed in Fig. 1.

The 93 studies reported on 77 publications and 372 unique associations between a SES indicator and PA outcome. The majority of the studies were conducted in Europe (54), followed by North America (14), Asia (14), South America (4), Africa (4), and Australia (3) (Table 2).

The sample size ranged from small studies with 131 participants to multi-country studies with sample sizes of up to 10.190 participants. Most studies reported a response rate between 60% and 80%. 35 publications did not report response rates, and 6 studies reported partial or incomplete information. Most studies (64) used self-reports (questionnaires) to assess PA. The most frequently validated PA questionnaire was the Physical Activity Questionnaire for Older Children (PAQ-C) [42]. Nineteen studies applied objective measurement methodology. The studies predominantly used accelerometers (ActiGraph GT3X, GT3X+, GT1M). Fourteen studies used both methods. Most of the studies were single studies. However, some studies were from more extensive cohort studies, e.g. ISCOLE Study, ENERGY-project, Childhood Obesity Surveillance Initiative (COSI), and Millennium Cohort Study (MCS). Concerning the SES indicators, the most prominent indicator was education, followed by occupation and income. Only 2 studies have calculated an SES index based on education, occupation, and income. Relating PA, most of the studies focused on MVPA and TPA, followed by OPA and LTPA. Only a few studies measured VPA, LPA, and MPA (Table 3).

### Education

Results showed a strong association between education and OPA, with nearly all studies demonstrating that higher education led to increased levels of organized PA participation in uni- and multivariate models (Tables 7 and 8). Studies only applied subjective methods of PA measurement. Overall, for education and TPA, most studies showed no association, which was supported by the multivariate study results. A more detailed analysis demonstrated a negative association between objectively measured TPA and maternal education when both genders were combined (2/3 studies) and in gender-separated analysis (*n* = 1 for boys and girls, respectively). For subjective assessments of TPA, there was a difference between gender-combined and separated studies, with no or negative associations in gender-combined analyses, no association for girls and no or positive associations in boys. Similarly, for MVPA, the vast majority of studies found no associations with education, with very few studies providing gender-separated analyses for paternal (*n* = 1) and maternal (*n* = 2) education (Table 4). Trials on maternal education found inconsistent results, with one study each demonstrating no or negative associations for boys and girls when measured objectively, an inconsistency also displayed when genders were combined. Contrarily, for paternal education, a positive association was found in girls; again, MVPA was measured objectively. No association could be identified for boys or when genders were combined. All seven gender-combined studies showed no association when MVPA was measured subjectively, while for maternal (*n* = 3) and paternal (*n* = 2) studies, positive and negative associations were found. When measured objectively, nearly all studies did not find associations, with few demonstrating negative ones. Using parental education combined, the results draw a similar picture to maternal education. Multivariate studies were somewhat similar to the results of objectively measured MVPA trials (Tables 5 and 6).

Few studies assessed LPA and MPA, all applying objective measures. Overall, most studies showed no associations between these PA measures and education (Table 4). Due to the lack of studies, no information is available for genders about paternal and maternal education. One of two studies found a negative association between MPA and maternal education when genders were analyzed together. For LPA and parental education mixed, there was no association when genders were combined, while for both genders separated (*n* = 2 each), inconsistent results with no or negative associations were found. The multivariate results confirmed the negative association for both genders. For VPA, inconsistent results were identified. While most studies showed no associations with education, two trials using objective measurements found positive associations. A Swedish representative sample demonstrated that girls with higher-educated parents were more vigorously active than their less-educated counterparts [43]. Similarly, one of two studies using maternal education as SES indicator found a positive association. While objectively measured, there was no negative association (*n* = 3), and this was the case in more than one-third of the subjectively reported studies (*n* = 7/19) for which only three found positive associations.

Few studies investigated the relationship between education and LTPA, and all of them applied subjective PA methods. Just one and two studies on paternal and maternal education respectively separated analyses by gender and found no association (Table 6). Two of three (all multivariate) studies found negative associations for this PA outcome for both paternal and maternal education when genders were combined. When parental education was combined as were genders, heterogeneous results were reported, with some studies showing positive, no, or negative associations. When looking at gender-separated analyses, no positive associations were found (Table 7).

### Occupation

For the association of occupation and OPA, only self-report studies were included (Table 6), and all applied multivariate analyses (Table 7). Results demonstrate no effect of maternal (*n* = 1) and paternal (*n* = 1) occupation. The same result was found in about one-third of studies combining parental occupation. Here, the majority pointed to a positive relationship. For TPA, no association was found in any study for combined parental, maternal, and paternal occupation. Regarding MVPA, most studies found no association with few reporting a negative relationship between MVPA and occupational status for maternal and paternal occupation. There was no study demonstrating a positive association. For VPA and MPA, only self-report studies were available, showing no associations between parental occupation combined and maternal occupation. No data were reported on paternal occupation (Table 4). For VPA, three out of 18 studies found negative associations for children in Latvia, Albania, and Spain, while the other studies found none. No data were available for LPA. For LTPA, parental occupation combined was positively associated for boys and girls separately, while no such relationship was identified using maternal or paternal occupation. However, for these measures, only one study each was found. When genders were combined, no association was found for parental and maternal occupation. For paternal occupational status, overall one in three or one in two (multivariate analysis) found positive associations (Tables 4 and 7).

### Income

Only self-report studies were identified for the association of income and OPA (outcome). Overall, a strong positive association exists between household income and this outcome in studies that combined both genders. Conversly, contrary results were reported for both genders (*n* = 2 each). With regard to TPA, there appears to be no association with income (Table 6). In multivariate analyses, neither boys’ (*n* = 2) nor girls’ (*n* = 2) TPA was affected by the respective household income, measured both objectively and by self-report. Few studies in gender-combined analyses and one study in girls pointed towards a positive association, while one large, representative sample in the US found a negative association. There are inconsistent results for MVPA with regard to household income, with studies mostly split between no and negative associations. Only objective studies (*n* = 2 for both genders) were available for gender-separated analyses. Multivariate analyses came to the same results (Table 7). For LPA, MPA, and VPA, few available studies overall showed partially positive associations for VPA (*n* = 1/2), somewhat negative associations for MPA (*n* = 1/2), and the one study reporting on this outcome found a negative association between income and LPA (Table 4). No study reported results separated for boys and girls. For objectively measured LTPA, no studies could be identified (Table 5). The few studies showed no associations between income and LTPA in gender-separated analyses. In studies reporting both genders combined, inconsistent results between studies were reported, all of which applied multivariate methods.

### SES

The few studies investigating the relationships between SES and subjectively measured PA found a positive association for TPA, while no associations could be identified for MVPA and LTPA (Tables 4, 6, 7 and 8).

## Discussion

The primary purpose of this study was to systematically review the recent evidence about the associations between family socioeconomic indicators (education, occupation, and household income and their combination (SES)) in different domains (e.g., OPA) or intensities of PA among primary school-aged children and to quantify these associations.

Overall, the analyses showed great heterogeneity in terms of outcomes, measurement of PA and SES variables, and often no clear effects contrary to what has been proclaimed in the literature. A relatively straightforward relationship existed between all individual socioeconomic factors and OPA. Results showed a predominantly positive relationship consistent across all gender variants (maternal, paternal, and parental), indicating that children from mothers and fathers with a higher education, occupation, and income have a higher probability for OPA. This association is likely related, among other things, to monetary conditions. OPA in an institution (e.g., a sports club) costs money, therefore children of higher-income households have fewer financial barriers to participation in OPA than those with a lower household income [44–47]. Strategies to reduce the gap between higher and lower-income families regarding participation and dropout rates in organized physical activities for their children need to focus on the reduction of financial barriers, e.g., through the use of vouchers [48–50].

In addition to the financial requirements, the parents’ educational background is a relevant factor for children’s participation in OPA. Parents with a higher level of education are more likely to deal with topics such as health behavior and to understand the significance of insufficient PA. As a consequence, higher educated parents more often act as role models for their children by being physically active themselves, and they are also more likely to be involved in OPA for their children (e.g., transportation) [51–54].

In contrast, the results for the different intensities of daily PA (TPA, LPA, MPA, MVPA, VPA, LTPA) are very heterogeneous. Regarding the intensities, an imbalance was shown in terms of the frequencies. Most studies were focused on MVPA. This is probably because the evidence and study situation for the association of MVPA with health benefits is better than for other PA intensities. Existing international recommendations focus on MVPA [55], which in turn causes this intensity being most frequently studied. In addition to MVPA, there are also relatively clear recommendations and evidence for VPA, addressed in many studies as part of the WHO European Childhood Obesity Surveillance Initiative (COSI) [56]. After MVPA and VPA, some studies addressed leisure-time PA. A notable aspect here was the sum of different terminology (e.g., free play, outdoor play, after-school PA, weekend, etc.) for LTPA. Therefore, a clear delimitation from other PA intensities or domains was difficult at some points. Fewest studies were found for TPA, LPA, and MPA. For these PA intensities, however, there is little research and thus no clear recommendations.

Contrarily, the results for associations between parental education and unorganized PA (especially outdoor play) were rather heterogeneous. Most of the PA intensities (TPA, MVPA, VPA) did not show associations or rather negative ones, especially for maternal education and for girls. There were a few outliers with tendencies toward a positive association for boys and for paternal education. Similar findings were reported previously [57, 58]. Possible explanations could include the educational trajectories of children from families with higher levels of education. Higher forms of schooling usually also mean a higher workload with school tasks (e.g., longer school days, more demanding homework) and thus less free time available for PA.

Regarding the occupational status of parents, the majority of studies showed no association and, if anything, a tendency toward a negative association. The same tendency could be found for income. However, studies on the SES index have shown positive or no association. As only two publications of one longitudinal study from Germany were identified for such an index and only associations for a few PA domains were reported, the certainty of this association is very low.

Most studies used parental education, followed by parental occupation, and just a few studies used parental or household income as a marker of SES, likely because information about the level of education is considered less sensitive compared to other information related to SES. Therefore, the response rate is relatively high compared to income. The least of all were studies that used a SES index. This is interesting because most studies reported on the influence of SES or socioeconomic position, mostly measuring only one variable and not having multiple pieces of information to calculate an actual complex index. However, there are differences between the various SES indicators, and with the above-average number of studies that only included education as an SES indicator, a bias in the overall picture of the relationships could potentially arise. To provide a complete picture of the socioeconomic situation, a comprehensive index should be collected [59].

Furthermore, results demonstrate an imbalance in gender-specific data of the individual SES indicators. More studies reported associations between maternal SES indicators, e.g., maternal educational level. This is probably because mothers, compared to fathers, are more responsible for organizational tasks related to the child [32], including filling in questionnaires for their children. Therefore, there is a lack of information on paternal SES indicators, reducing power and certainty into the evidence for latter. These findings differ for income as information on this parameter is usually inquired on a household level, which means that information on both parents’ income is integrated.

Some general aspects are limiting the comparability of identified studies on the one hand, but on the other hand, can also explain the heterogeneity of the results. The location and timing of data collection are essential to consider because of weather and seasonal influences, especially for unstructured PA (e.g., outdoor play) [60–62]. This may lead to different results by collecting and analyzing the same parameters. But also different ethnic backgrounds under which the studies took place lead to different results [63, 64] and make international comparability difficult. Also, environmental and structural conditions play a role in the heterogeneity of the results. For example, children from families with similar SES backgrounds but different living situations (urban versus rural) have different PA behaviors [65], e.g., the access for children from rural areas to sports clubs may be limited. Overall, the generalizability of the results on a global level is potentially limited as the majority of the included studies were conducted in Europe.

The heterogeneity of the associations of SES factors, especially in unorganized PA, illustrates the complexity of this context. This was also shown by non-linear and U-shaped associations in some studies. However, fewer studies are analyzing multivariate models for the relationship between SES and its single factors and PA in which various relevant factors (including mediators and moderators) are considered. However, a multivariate approach is better since univariate models do not meet the requirements of the complexity of the topic. Future research in this area should take this into account and adjust for multiple relevant factors.

### Strengths and limitations

A strength of this review is the clustering of the identified studies into several smaller subcategories (e.g., objective vs. subjective, univariate vs. multivariate, gender-combined vs. separated) and, by that enabling a more specific insight into this complex topic. We identified several research gaps, such as the need for more information on paternal SES indicators.

Overall, it is important to point out that socioeconomic disparities in health represent a fundamentally complex area of research due to the intricate causal mechanisms involved. The predominance of evidence derived from observational studies introduces the risk of confounding bias, which is defined as a spurious association introduced by an extraneous variable that influences both the socioeconomic exposure and the health outcome [66]. Moreover, due to this complexity, there is also a risk of overadjustment [67]. Considering these factors, the current review describes associations but cannot clearly demonstrate causal relationships.

Furthermore, several other limitations need to be acknowledged. First, PA measures, such as VPA or MVPA, may have been taken during an organized activity in a sports club. Thus, classifying unstructured PA (e.g., free play) or organized PA is complicated or overlapping. This point concerns objective measures more than collecting incidental and planned activities, while self-report measures gather mostly larger chunks of habitual PA information. Second, the possibility of different coding and categorization of the SES indicators may have affected the results. For example, occupational status may have been collected dichotomously (yes or no), according to the number of hours (full-time, part-time, marginal) or according to the actual activity (e.g., craft sector or office) or position (e.g., leading position) and then categorized into high, medium or low. Also, different cut-off values for the SES indicators complicate comparisons between studies (e.g., different thresholds for categorizing the educational level as high, medium, and low). Third, only individual SES indicators were considered in this study. Thus, all studies collecting SES based on the living area (e.g., via Zipcode) were not considered. However, compared to area-based SES measures, SES obtained individually shows greater associations with health outcomes and avoids masking significant heterogeneity amongst populations [68]. In addition, as we focused on few common SES indicators as well as the SES index, it was discovered that children and adolescents, in particular, were unable to provide substantial responses regarding their families’ finances (e.g., paternal occupation) [69]. Consequently, less intrusive and more comprehensible approaches, such as the Family Affluence Scale (FAS), are also used to determine their socioeconomic status. However, this is more prevalent in older children and adolescents. In the age cohort under consideration, it can be assumed that the questionnaires are completed by the parents and that the relevant information can be provided in a valid manner. Nevertheless, the incorporation of additional alternative scales for the assessment of SES (such as the FAS) in future studies could prove beneficial in further elucidating this matter. Lastly, due to the large heterogeneity in the measurement of the SES variables and the study populations (e.g., studies in different regions, at different time points, with different ethnic groups, and with different initial questions), we decided against performing a meta-analysis [70]. Future evidence syntheses may investigate specific factors and attempt to pool results mathematically.

## Conclusion

The results of this review showed predominantly positive associations between the individual socioeconomic factors education, occupation and income, and organized PA. In contrast, the results for SES indicators and different intensities of daily PA (TPA, LPA, MPA, MVPA, VPA, LTPA) were very heterogeneous, with overwhelmingly no associations. An uneven distribution was shown with most studies measuring maternal education as a benchmark for family SES. Overall, there is a lack of large multicenter studies using an accurate SES index as a predictor. Future research should focus on larger multicenter studies and analyzing gender-specific multivariate models for the relationship of SES and especially children’s unstructured PA, considering potentially relevant mediators and moderators to cover the complexity of the association.

### Electronic supplementary material

Below is the link to the electronic supplementary material.

Supplementary Material 1

## Data Availability

Data will be available upon request of the corresponding author.

## Abbreviations

PA: Physical Activity
TPA: Total physical activity
LTPA: Light physcial activity
MPA: Moderate physical activity
MVPA: Moderate-to-vigorous physical activity
VPA: Vigorous physical activity
LTPA: Leisure-time physical activity
OPA: Organized physical activity
AT: Active transport
SES: Socioeconomic status
PRISMA: Preferred Reporting Items of Systematic Reviews and Meta-Analysis
