# Supplementary material for: Associations of family socioeconomic indicators and physical activity of primary school-aged children: a systematic review
Source: BMC Public Health. 2024 Aug 19;24:2247. doi: 10.1186/s12889-024-19174-6 (PMC11331658; doi:10.1186/s12889-024-19174-6)
Supplement: Supplementary file 1 — Supplementary Material 1 [file 12889_2024_19174_MOESM1_ESM.pdf]

Risk of Bias assessment for the included studies

Legend

	Response rate	Adjustment	Sample size
Green	≥ 50%	adjusted for at least one variable (e.g. age, gender, etc.)	≥ 500
Yellow	unclear / not provided / unspecified	unclear / not provided / unspecified	unclear / not provided / unspecified
Red	< 50 %	not adjusted	< 500

Study	Response rate	Adjustment	Sample size
(Aarts et al., 2012)	Green	Green	Green
(Aarts et al., 2010)	Green	Green	Green
(Aggio et al., 2017)	Yellow	Green	Green
(Aguilar-Farias et al., 2019)	Green	Green	Red
(Al Yazeedi et al., 2021)	Yellow	Green	Red
(Alotaibi et al., 2020)	Green	Yellow	Red
(Atkin et al., 2016)	Green	Green	Green
(Bagordo et al., 2017)	Green	Yellow	Green
(Barr-Anderson et al., 2017)	Green	Red	Green
(Beckvid Henriksson et al., 2016)	Green	Green	Green
(Brug et al., 2012)	Green	Green	Green
(Butte et al., 2014)	Yellow	Green	Red
(Cadogan et al., 2014)	Green	Green	Green
(Cárdenas-Fuentes et al., 2021)	Yellow	Green	Green
(Cvetković et al., 2014)	Yellow	Yellow	Green
(da Silva et al., 2014)	Green	Yellow	Green
(de Moraes Ferrari et al., 2016)	Green	Yellow	Red
(Deng & Fredriksen, 2018)	Green	Yellow	Green
(Ding et al., 2020)	Yellow	Green	Green
(Dmitruk et al., 2015)	Green	Yellow	Red
(Drenowatz et al., 2010) (Study 1)	Green	Green	Red
(Drenowatz et al., 2010) (Study 2)	Green	Green	Red
(Duncan et al., 2012)	Yellow	Yellow	Green
(Engel-Yeger, 2012)	Yellow	Green	Red
(Fakhouri et al., 2013)	Yellow	Yellow	Green
(Fernández-Alvira et al., 2015)	Yellow	Green	Green
(Gomes et al., 2017)	Green	Green	Red
(Harbec et al., 2021)	Green	Green	Green
(Herzig et al., 2012)	Red	Green	Green
(Huang et al., 2010)	Green	Green	Green
(Huang et al., 2013)	Green	Green	Red
(Janssen et al., 2014)	Green	Green	Green
(Jerina et al., 2018)	Yellow	Yellow	Green
(Jiménez-Pavón et al., 2012)	Green	Green	Green
(Kawalec & Pawlas, 2021)	Yellow	Green	Red
(Knuth et al., 2017)	Green	Green	Green
(Kobel et al., 2015)	Yellow	Green	Green
(Lämmle et al., 2012)	Green	Yellow	Green
(Lampinen et al., 2017)	Yellow	Green	Red

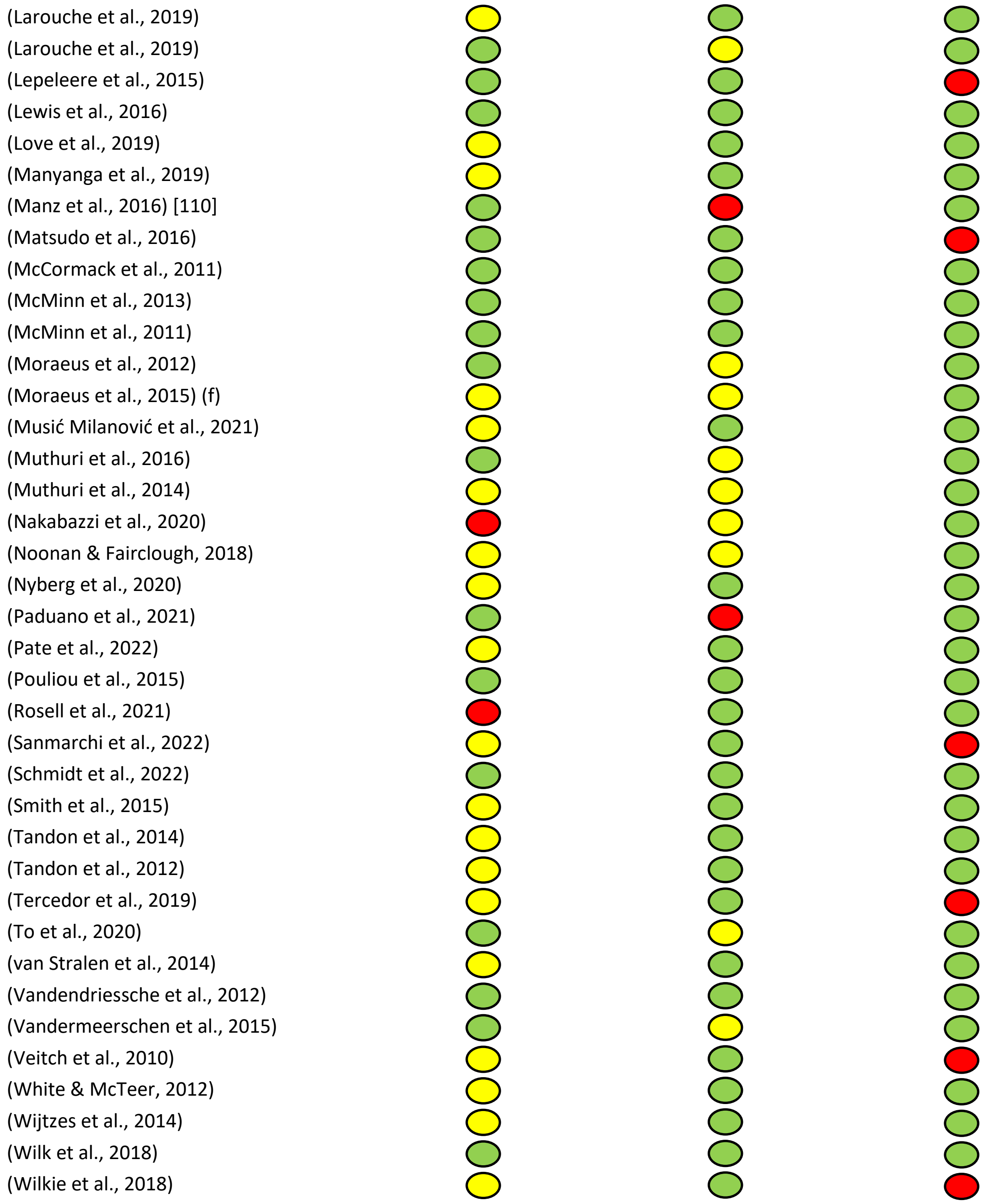
(Larouche et al., 2019)
(Larouche et al., 2019)
(Lepeleere et al., 2015)
(Lewis et al., 2016)
(Love et al., 2019)
(Manyanga et al., 2019)
(Manz et al., 2016) [110]
(Matsudo et al., 2016)
(McCormack et al., 2011)
(McMinn et al., 2013)
(McMinn et al., 2011)
(Moraeus et al., 2012)
(Moraeus et al., 2015) (f)
(Musić Milanović et al., 2021)
(Muthuri et al., 2016)
(Muthuri et al., 2014)
(Nakabazzi et al., 2020)
(Noonan & Fairclough, 2018)
(Nyberg et al., 2020)
(Paduano et al., 2021)
(Pate et al., 2022)
(Pouliou et al., 2015)
(Rosell et al., 2021)
(Sanmarchi et al., 2022)
(Schmidt et al., 2022)
(Smith et al., 2015)
(Tandon et al., 2014)
(Tandon et al., 2012)
(Tercedor et al., 2019)
(To et al., 2020)
(van Stralen et al., 2014)
(Vandendriessche et al., 2012)
(Vandermeerschen et al., 2015)
(Veitch et al., 2010)
(White & McTeer, 2012)
(Wijtzes et al., 2014)
(Wilk et al., 2018)
(Wilkie et al., 2018)
